# Interface Engineering of CoS/CoO@N-Doped Graphene Nanocomposite for High-Performance Rechargeable Zn–Air Batteries

**DOI:** 10.1007/s40820-020-00526-x

**Published:** 2020-10-27

**Authors:** Yuhui Tian, Li Xu, Meng Li, Ding Yuan, Xianhu Liu, Junchao Qian, Yuhai Dou, Jingxia Qiu, Shanqing Zhang

**Affiliations:** 1 Institute for Energy Research, School of Chemistry and Chemical Engineering, Key Laboratory of Zhenjiang, Jiangsu University, Zhenjiang, 212013 People’s Republic of China; 2 Centre for Clean Environment and Energy, School of Environment and Science, Gold Coast Campus, Griffith University, Gold Coast, Queensland 4222 Australia; 3 Key Laboratory of Materials Processing and Mold (Zhengzhou University), Ministry of Education, Zhengzhou, People’s Republic of China; 4 Jiangsu Key Laboratory for Environment Functional Materials, Suzhou University of Science and Technology, Suzhou, 215009 People’s Republic of China

**Keywords:** Cobalt sulfide/oxide, Heterostructure, Interface, Bifunctional electrocatalyst, Rechargeable Zn–air battery

## Abstract

Low cost and green fabrication of high-performance electrocatalysts with earth-abundant resources for oxygen reduction reaction (ORR) and oxygen evolution reaction (OER) are crucial for the large-scale application of rechargeable Zn–air batteries (ZABs). In this work, our density functional theory calculations on the electrocatalyst suggest that the rational construction of interfacial structure can induce local charge redistribution, improve the electronic conductivity and enhance the catalyst stability. In order to realize such a structure, we spatially immobilize heterogeneous CoS/CoO nanocrystals onto N-doped graphene to synthesize a bifunctional electrocatalyst (CoS/CoO@NGNs). The optimization of the composition, interfacial structure and conductivity of the electrocatalyst is conducted to achieve bifunctional catalytic activity and deliver outstanding efficiency and stability for both ORR and OER. The aqueous ZAB with the as-prepared CoS/CoO@NGNs cathode displays a high maximum power density of 137.8 mW cm^−2^, a specific capacity of 723.9 mAh g^−1^ and excellent cycling stability (continuous operating for 100 h) with a high round-trip efficiency. In addition, the assembled quasi-solid-state ZAB also exhibits outstanding mechanical flexibility besides high battery performances, showing great potential for applications in flexible and wearable electronic devices.

**Electronic supplementary material:**

The online version of this article (10.1007/s40820-020-00526-x) contains supplementary material, which is available to authorized users.

## Highlights

- Interface engineering of heterogeneous CoS/CoO nanocrystals and N-doped graphene composite facilitates high-performance oxygen reduction reaction and oxygen evolution reaction.
- Density functional theory calculations and experimental results confirm the enhanced electrocatalytic performances via the proposed interface engineering.
- The bifunctional oxygen electrocatalyst exhibits excellent performances in rechargeable Zn–air batteries.

**Electronic supplementary material:**

The online version of this article (10.1007/s40820-020-00526-x) contains supplementary material, which is available to authorized users.

## Graphical abstract

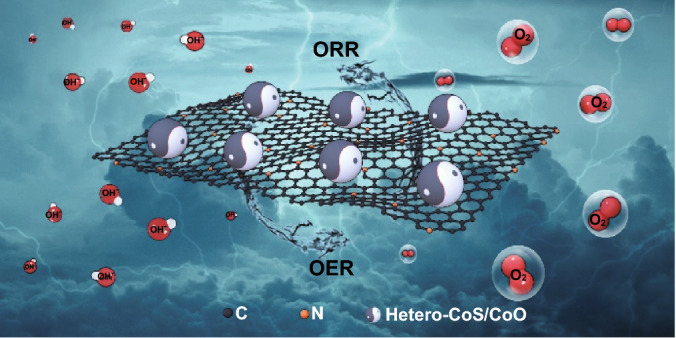

## Introduction

The advanced electrocatalysis technologies, such as water splitting, fuel cell and metal-air battery, have received increasing attention in the development of next-generation clean and sustainable energy devices [1]. The rechargeable Zn–air batteries (ZABs) are one of these new energy conversion and storage systems with great potential for large-scale applications due to its high theoretical power density, natural abundance of Zn, environmental friendliness, safety and low cost [2–4]. In rechargeable ZABs, oxygen reduction reaction (ORR) and oxygen evolution reaction (OER) are two critical electrochemical reactions during the discharge and charge operation, respectively [5]. The practical performance of rechargeable ZABs is significantly influenced by the kinetics of these two reactions. However, both ORR and OER involve a multistep proton–electron transfer and suffer from high energy barriers and sluggish reaction kinetics, which lead to low energy efficiencies and high overpotentials [6, 7]. Therefore, developing high-performance and low-cost bifunctional electrocatalysts, capable of simultaneously catalyzing ORR and OER, is of great importance for the large-scale application of rechargeable ZABs [8].

Generally, there are several requirements for optimal electrocatalysts, including rational chemical compositions and electronic structures for reduced reaction energy barriers and enhanced reaction rates [9], high electron conductivity for rapid kinetics [10], excellent stability for long-term operation [11] and low-cost and earth-abundant resources for scalable manufacturing and commercialization [12]. To meet these requirements, significant efforts have been devoted to material design and engineering. It is well established that the bond breakage of reactants, the bond formation of intermediates and products, and the electron transfer generally occur at the active sites of a catalyst [13]. Active sites located at the interface with high intrinsic activity can lead to significantly enhanced electrocatalytic performances [14]. As such, it is important to design the interfacial structure to achieve the desired catalytic functions. According to previous theoretical and experimental investigations, the heterostructure-induced interfacial charge transfer and coupling effects between different components have the potential to yield high performances and novel functionalities in electrocatalysis [15, 16]. On the other hand, the structural discontinuities and interfacial dislocations induced by the interfacial structure can also be exploited to improve the surface binding energy with intermediate species during electrocatalysis, leading to improved electrocatalytic activity [17, 18]. In this regard, engineering the interfacial structure of heterogeneous catalysts can be regarded as an effective strategy for the rational design of bifunctional ORR/OER electrocatalysts.

In heterogeneous catalysts, there are two typical interfacial structures: the boundary between different components and the interface between active species and their support matrix [14]. For boundary interfaces, various interfacial structures, such as metal/oxide [16], oxide/oxide [18], sulfide/sulfide [19] and carbide/nitride [20], have been reported to exhibit enhanced ORR/OER performances. As non-precious and earth-abundant materials, Co-based sulfides and oxides are promising candidates to construct heterostructure catalysts due to their component variety, structural adjustability, facile preparation and considerable bifunctional ORR/OER activity [21]. On the other hand, anchoring metal species on conductive supports gives rise to an additional interface in heterogeneous electrocatalysts. Since catalyst supports play an important role in immobilizing active species, controlling their spatial distribution and enhancing their conductivity and stability, engineering the interaction between active materials and their supports has a significant impact on the electrocatalytic property [22]. Carbon-based materials can serve as an excellent matrix to support metal species due to their high surface area and structural stability [23]. Particularly, N-doped carbon materials have emerged as rising-star substrates for constructing electrocatalysts because N-doping can alter the electronic structure of pristine carbon frameworks and make them efficient for anchoring and stabilizing metal species [24, 25]. Moreover, the excellent electronic conductivity of N-doped carbon can accelerate electron transfer during the electrocatalysis process, further enhancing the catalytic performance [23].

Based on the above discussion, we rationally designed a nanocomposite comprising spatial immobilization of heterogeneous CoS/CoO nanocrystals onto N-doped graphene (CoS/CoO@NGNs) as the bifunctional electrocatalyst for both ORR and OER. Density functional theory (DFT) calculations demonstrate that the cobalt sulfide/oxide interfaces would bring enhanced electrical conductivity with efficient electron transfer. Meanwhile, the interfacial coupling effect between CoS/CoO and N-doped graphene can impart excellent stability of the catalyst. Electrochemical tests show that the synthesized CoS/CoO@NGNs catalyst displays high catalytic activity and excellent catalytic durability toward both ORR and OER. As a practical application, the aqueous and flexible quasi-solid-state ZABs with CoS/CoO@NGNs air electrodes present better cycling stability and higher energy efficiency than batteries with noble-metal Pt/C + IrO_2_ mixture catalysts. This work provides an effective and promising strategy to construct highly active and stable bifunctional oxygen electrocatalysts for enhancing rechargeable ZAB performances.

## Experimental

### Synthesis of CoS/CoO@NGNs

Precursors for Co(OH)_2_ nanosheets and N-doped graphene nanosheets (NGNs) were first synthesized (experimental section in the supporting information). Then, a total of 20 mg Co(OH)_2_ and 20 mg of NGNs were dispersed into two separate beakers with 10 mL of ethanol, respectively. After ultrasonic treatment for 30 min, the Co(OH)_2_ suspension was poured into the NGNs suspension. Then, 30 mg of thioacetamide was added into the mixture. After magnetic stirring for 30 min, the mixture was transferred into a Teflon-lined stainless-steel autoclave and heated at 160 °C for 4 h. After cooling to the room temperature, the resulting sample (denoted as CoS@NGNs) was collected by filtering and washed with deionized water and ethanol and then dried in a vacuum oven at 60 °C overnight. The controlling of oxygen concentrations plays crucial role on the oxidation degree of the CoS precursor. In oxygen-rich environment, the CoS could be oxidized to CoO and then could be over-oxidized to Co_3_O_4_. In order to obtain pure CoO, avoid the over-oxidation and to secure the oxygen-poor environment, we made use of the trace amount of oxygen in the commercial argon gas (with a purify of 99.99%) as the oxygen source. The CoS/CoO@NGNs catalyst was obtained by annealing CoS@NGNs with a ramp rate of 5 °C min^−1^ to 300 °C for 3 h under the argon atmosphere. Accompanied by the cooling down process in air. For comparison, the CoO phase-dominated CoO@NGNs catalyst was obtained by heating CoS@NGNs at the same condition for 12 h and then cooling down in the air.

### Electrochemical Measurements

All electrochemical measurements were taken on a CHI 760E electrochemical workstation (CH Instrument, USA) with a typical three-electrode system. A glassy carbon electrode (GCE) with a diameter of 3 mm and Ag/AgCl electrode (saturated KCl) was used as the counter electrode and the reference electrode, respectively. The test of ORR and OER catalytic activity was conducted on the rotating ring disk electrode (RRDE) with a diameter of 4 mm. To prepare the working electrode, 3 mg of the catalyst was dispersed into 1000 μL of 1:1 water/ethanol solution and 20 μL of Nafion solution (10 wt%), followed by ultrasonic treatment for 1 h. Then, 10 μL of the catalyst ink was loaded onto the surface of glassy carbon in RRDE. All the potentials were calibrated to reversible hydrogen electrode (RHE) according to the Nernst equation (*E*_RHE_ = *E*_Ag/AgCl_ + 0.059pH + 0.197).

For ORR tests, cyclic voltammetry (CV) tests were performed in O_2_ or N_2_-saturated 0.1 M KOH with a scan rate of 50 mV s^−1^. LSV curves were recorded with a scan rate of 10 mV s^−1^, and the capacitive current was eliminated. For RRDE tests, the ring electrode potential was set to 1.25 V (vs. RHE) to oxidize HO_2_^−^ from the disk electrode. The electron-transfer number and the percentage of HO_2_^−^ were calculated according to the following equations:

1 $$n = 4\left| {I_{\text{d}} } \right| / (\left| {I_{\text{d}} } \right|{ + }I_{\text{r}} /N )$$

2 $${\text{HO}}_{2}^{ - } = 200 \times I_{\text{r}} /(I_{\text{r}} + N\left| {I_{\text{d}} } \right|)$$

where *I*_d_ is the disk current, *I*_r_ represents the ring current, and *N* is the current collection efficiency of the Pt ring with a value of 0.37.

The electron-transfer number per oxygen molecule was also calculated from the Koutecky–Levich (K–L) equation:

3 $$\frac{1}{j} = \frac{1}{{j_{\text{k}} }} + \frac{1}{B}\omega^{ - 1/2}$$

4 $$B = 0.2\;{\text{nF}}(D_{{({\text{O}}_{2} )}} )^{2/3} v^{( - 1/6)} C_{{({\text{O}}_{2} )}}$$

$\omega$ is the angular velocity of the disk (rpm). $n$ is the electron-transfer number for ORR. $F$ is Faraday constant (96,485 C mol^−1^). $D_{{({\text{O}}_{2} )}}$ is the diffusion coefficient of O_2_ in 0.1 M KOH (1.9 × 10^−5^ cm^2^ s^−1^).$v$ is the kinematic viscosity (0.01 cm^2^ s^−1^). $C_{{({\text{O}}_{2} )}}$ is the bulk concentration of O_2_ in the solution (1.2 × 10^−6^ mol cm^−3^).

The OER tests were conducted in N_2_-saturated 0.1 M KOH. LSV curves were recorded with a scan rate of 10 mV s^−1^. Electrochemical impedance spectroscopy (EIS) measurements were taken by applying an AC voltage with 5 mV amplitude in a frequency range from 100 kHz to 0.1 Hz at the potential of 1.6 V (vs. RHE) in 0.1 M KOH. The double-layer capacitance (*C*_dl_) of catalysts was investigated on the basis of CV curves recorded at different sweep rates (2, 5, 10, 15, 20, 25 and 50 mV s^−1^) in the potential range 0.964–1.064 V (vs. RHE).

### Density Functional Theory Calculation

The density functional calculations were performed in the Vienna ab initio simulation packages (VASP), using the generalized gradient approximation (GGA) with Perdew–Burke–Ernzerhof (PBE) parameterization [26]. Owing to the strong on-site Coulomb repulsion between the *d* electrons of Co atoms, DFT + U method with U = 3.50 eV was used for Co atoms [27]. The CoS/CoO heterostructure model was constructed by CoS (100) plane and CoO (111) plane, consisting 48 Co atoms, 24 O atoms and 24 S atoms. A vacuum space of 15 Å in the *z*-direction was used to avoid the possible interaction between neighboring slabs. To balance the demanding computational cost, a 2 × 2 × 1 Monkhorst–Pack k-point mesh was used for geometry optimization [28]. During the geometry optimization, the energy change criterion was set to 10^−4^ eV, and the maximum force was 0.03 eV Å^−1^. The energy cutoff of the plane wave basis was set as 400 eV. A graphene layer with the doping of pyridinic N, pyrrolic N and graphitic N was built to present the N-doped graphene nanosheet. Bulk CoS and CoO slabs with 2 × 2 supercells were used for stimulation. To approximately stimulate the interaction between the CoS or CoO slab and N-doped graphene layer, the van der Waals correction with DFT-D3 method was used [29]. The interfacial binding energy (Δ*E*_b_) was calculated according to the following equation [15]:

5 $$\Delta E_{{_{\text{b}} }} = E_{\text{total}} - E_{\text{CoS/CoO}} - E_{\text{NG}}$$

where *E*_total_, *E*_CoS/CoO_ and *E*_NG_ represent the total energy of the hybrid structure, individual CoS or CoO slab and N-doped graphene layer, respectively.

### Aqueous Zn–Air Battery Assembly

The aqueous Zn–air battery tests were performed with a homemade Zn–Air cell. The catalyst with a loading of 1.0 mg cm^−2^ was coated on a porous carbon paper (with a geometric area of 1 cm^2^). A polished Zn plate was used as the anode. Carbon cloth and nickel foam served as the gas diffusion layer on the air cathode and current collector, respectively. A mixed solution of 6 M KOH with 0.2 M zinc acetate was used as the electrolyte. The polarization curves were recorded by linear sweep voltammetry with a sweep rate of 10 mV s^−1^. The specific capacity and energy density were calculated from the galvanostatic discharge results, normalized to the mass of consumed Zn. The cycling test was conducted on a CHI 760E electrochemical workstation at a current density of 10 mA cm^−2^ (10 min for discharge and 10 min for the charge in each cycle).

### Flexible Quasi-Solid-State Zn–Air Battery Assembly

A polished Zn plate was tailored into 1.5 × 3 cm^2^ (with excessive part for connection) as the anode. Catalyst-coated carbon cloth (1 × 2 cm^2^, catalyst loading = 1 mg cm^−2^) was used as the cathode. Cu mech was fixed on the cathode as the current collector. The gel polymer electrolyte was prepared as follows: polyvinyl alcohol (PVA, MW 19500, Aladdin) powder (1.0 g) was dissolved in 10.0 mL of deionized water at 95 °C under magnetic stirring until the solution became clear. Then 1.0 mL of 18.0 M KOH filled with 0.1 M zinc acetate was added and stirred at 95 °C for 1.0 h. The solution was frozen at −3 °C over 12 h and then thawed at room temperature. Subsequently, the obtained gel electrolyte was tailored to the size of 1.5 × 3 cm^2^ and placed between the anode and cathode. Finally, the battery was constructed after being sealed with the white breathable tape.

## Results and Discussion

### DFT Calculations

**Fig. 1:**
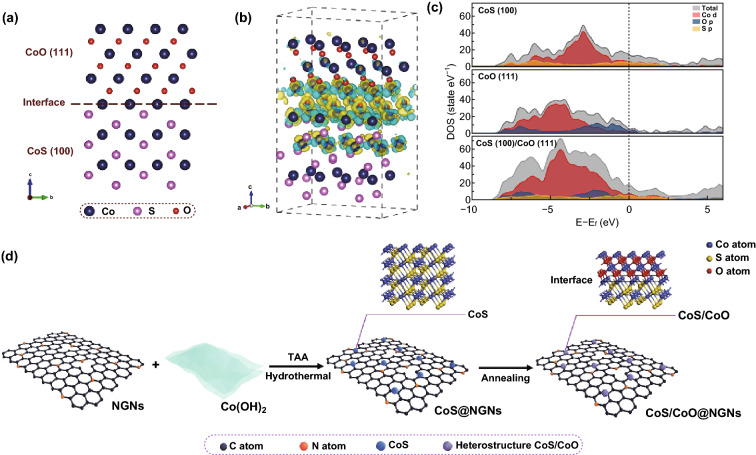
**a** Schematic of the CoS (100)/CoO (111) heterostructure model. **b** Contour plots of differential charge density of the CoS (100)/CoO (111) model. The yellow and cyan regions represent the charge accumulation and charge depletion, respectively. The iso-surface level was set to be 0.008 eÅ^−1^. **c** DOS plots of CoS (100), CoO (111) and CoS (100)/CoO (111). **d** Schematic illustration for the preparation of CoS/CoO@NGNs

As aforementioned, the boundary interfaces of a catalyst play an essential role in the bond breakage, bond formation, mass transport and electron transfer in an electrocatalytic reaction. For this, we firstly designed a CoS/CoO heterostructure model with the composition of CoS (100) and CoO (111) planes (Fig. 1a) and investigated its property via the DFT calculations. Charge density difference was obtained by subtracting the charge of the heterostructure from isolated CoS and CoO components. As shown in Fig. 1b, the charge redistribution can be observed at the interface between CoS and CoO, with the electron accumulation at the CoO side. Furthermore, Fig. 1c shows the increased density of states of the heterostructure near the Fermi level compared with the CoS (100) and CoO (111), which results from the hybridization of O, S p-orbital and Co d-orbital. The increased electron-occupied states at the Fermi level suggest the metallic feature of CoS/CoO heterostructure with enhanced intrinsic conductivity, which can improve the electrocatalytic performance via efficient electron transfer between catalyst surface and absorbed intermediates [30].

As for the interface between active species and substrate materials, a graphene layer with the doping of pyridinic N, pyrrolic N and graphitic N was used as the substrate to stabilize CoS/CoO. We built CoS-NG and CoO-NG models with bulk CoS and CoO attached above the N-doped graphene layer (NG), respectively (Fig. S1, S2). Figure S3 illustrates the existence of the charge accumulation region at the contact interface in both cases. Such electron accumulation at the metal–support interface can further associate to reduce the contact resistance and enable the superior electron-transfer ability of the catalyst, hence increasing the electrocatalytic kinetics [31, 32]. The interfacial binding energy (Δ*E*_b_) is calculated to be − 8.52 eV for CoS-NG and − 7.14 eV for CoO-NG, respectively. The thermodynamically feasible value of interfacial binding energy enables strong contact between supported metal species and the substrate, which is expected to result in better stability of the hybrid catalyst [25]. These calculation results theoretically reveal that constructing the interfacial structure in heterogeneous catalysts can impose a positive effect on enhancing electrocatalytic performance by offering improved conductivity, accelerated electron transfer and excellent stability.

### Physicochemical Characterizations

Guided by the theoretical predictions, we realized the interfacial structures comprising spatial immobilization of heterogeneous CoS/CoO nanocrystals onto N-doped graphene through experiments. Figure 1d briefly illustrates the preparation of this heterogeneous catalyst (see details in Experimental Section). First, CoS supported on N-doped graphene was prepared via a simple sulfurization process of Co(OH)_2_ nanosheets on N-doped graphene nanosheets with the assistance of thioacetamide. The precursors were evidenced by X-ray diffraction (XRD) and field-emission scanning electron microscopy (FESEM) in Figs. S4, S5. The as-prepared catalyst is denoted as CoS@NGNs. As illustrated in Fig. S6, after the hydrothermal treatment, 2D-structured Co(OH)_2_ nanosheets are converted into small CoS nanocrystals and in situ anchored on the surface of NGNs. Then, the catalyst with CoS/CoO interfacial structure (CoS/CoO@NGNs) was achieved via the annealing treatment, where the transformation of CoS to CoO occurred due to the small formation energy of CoO [33]. To elucidate the importance of dual-phase interfacial structure, the CoO phase-dominated CoO@NGNs catalyst was also fabricated for comparison (Fig. S7).

**Fig. 2:**
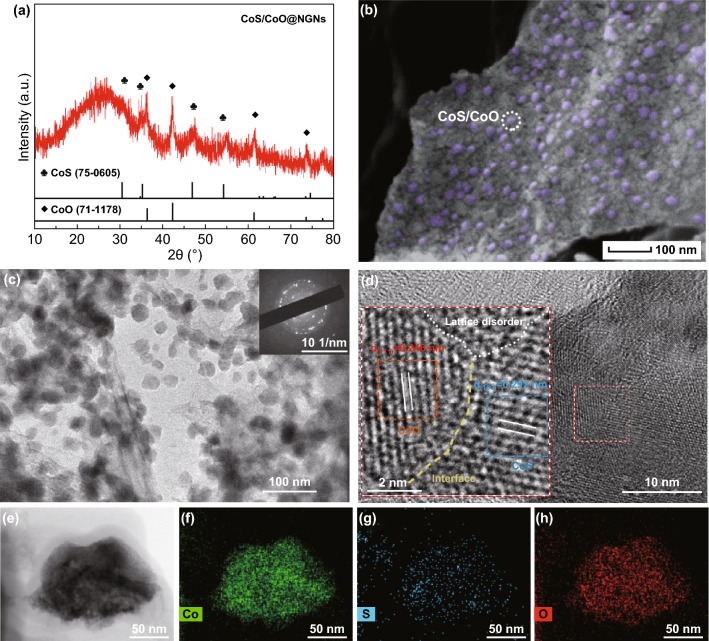
**a** XRD pattern, **b** SEM, **c** TEM (inset: SAED pattern) and **d** HRTEM images of CoS/CoO@NGNs (inset: the magnified image of the selected area). **e–h** High-angle annular dark-field scanning TEM (HAADF-STEM) image and corresponding EDS elemental maps of Co, S and O in CoS/CoO@NGNs

The heterostructure feature of CoS/CoO@NGNs was first identified by XRD. As shown in the XRD pattern of CoS/CoO@NGNs (Fig. 2a), typical diffraction peaks related to CoS and CoO can be observed. Specifically, the diffraction peaks at 30.5°, 35.1°, 47.2° and 54.3° can be assigned to the (100), (002), (101) and (110) planes of CoS (JCPDS No. 75-0605) [34]. The peaks at 36.5°, 42.5°, 61.8° and 73.6° can be indexed to the (111), (200), (220) and (311) planes of CoO (JCPDS No. 71-1178) [35]. In addition, a characteristic diffraction peak at 26° corresponds to the (002) facet of graphitic carbon [36]. No additional peak is observed, confirming the formation of mixed phases of CoS and CoO in CoS/CoO@NGNs.

The SEM image in Fig. 2b reveals that CoS/CoO@NGNs exhibits a much rougher surface in comparison with the pristine NGNs (Fig. S5). The well-dispersed cobalt-based nanocrystals (highlighted in purple) are anchored on the surface of NGNs. Such a structural feature is also reflected in the transmission electron microscopy (TEM) image of CoS/CoO@NGNs (Fig. 2c). The selected area electron diffraction (SAED) pattern (inset of Fig. 2c) demonstrates the polycrystalline feature of CoS/CoO@NGNs originated from different crystal phases of CoS and CoO. In the high-resolution TEM (HRTEM) image, the identified lattice distances of 0.292 and 0.246 nm match well with the (100) plane of CoS and (111) plane of CoO, respectively (Fig. 2d and inset) [37, 38]. A clear interface derived from two different domains (denoted by the yellow dash line) provides solid evidence for the existence of interfacial structure between CoS and CoO. Moreover, the lattice disorder (denoted by white dot line) can be observed. Such disordered structure can reduce the surface energy and result in more active sites, leading to promoted electrocatalytic performances of the catalyst [19, 39]. The energy-dispersive X-ray spectroscopy (EDS) elemental mapping images clearly show that S and O signals are concentrated at the Co signal (Fig. 2e–h). The electron energy loss spectroscopy (EELS) line profile acquired on a single nanocrystal in CoS/CoO@NGNs demonstrates the asymmetric distribution of S and O elements along the whole CoS/CoO nanocrystal (Fig. S8), further suggesting the formation of heterointerfaces between CoS and CoO.

The N_2_ adsorption–desorption isotherms of NGNs and CoS/CoO@NGNs present typical type-VI curves with a distinct hysteresis loop, indicating the presence of split mesopores resulting from the stacking sheet-like structure (Fig. S9) [40]. The specific surface area is 684.6 m^2^ g^−1^ for NGNs. After incorporation of cobalt-based nanocrystals, the specific surface area of CoS/CoO@NGNs shrinks to 89.0 m^2^ g^−1^. The N-doped graphene with high conductivity will bring about enhanced electron transfer, and the spatial dispersion CoS/CoO nanocrystals on the surface can reduce the re-stacking of N-doped graphene nanosheets, ensuring highly accessible surface areas of the catalyst [32]. As a result, more active sites can be exposed through this interfacial structure, hence enhancing the electrochemical activity.

**Fig. 3:**
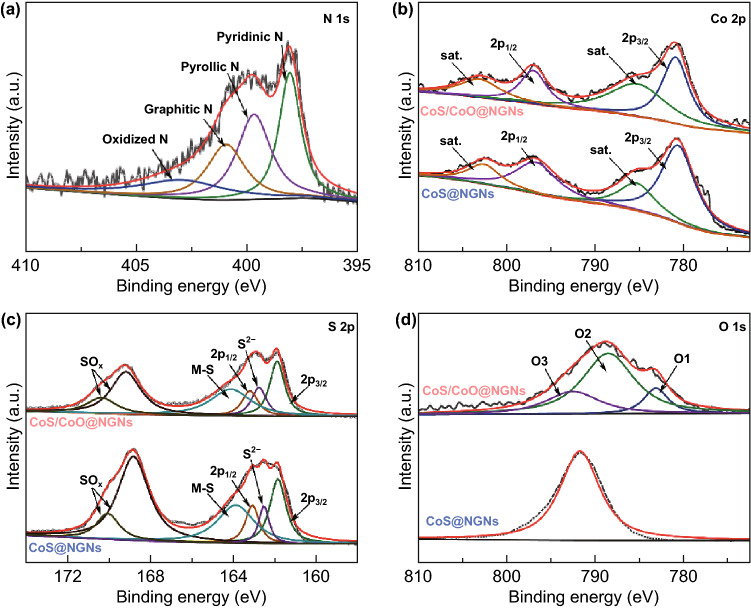
**a** N 1 s XPS spectrum of CoS/CoO@NGNs. **b** Co 2p, **c** S 2p and **d** O 1 s XPS spectra of CoS@NGNs and CoS/CoO@NGNs

X-ray photoelectron spectroscopy (XPS) analyses were performed to investigate the elemental compositions and surface chemical states of CoS/CoO@NGNs. The survey spectrum confirms that the CoS/CoO@NGNs catalyst is composed of S, C, N, O and Co elements (Fig. S10). In the N 1 s high-resolution spectrum (Fig. 3a), four deconvoluted subpeaks at 398.1, 399.7, 401.0 and 402.9 eV belong to pyridinic N, pyrrolic N, graphitic N and oxidized N, respectively [41, 42]. Doping N atoms into the carbon framework can induce a relatively high positive charge density on the adjacent carbon [43], which not only contributes to the enhancement of ORR activity [44], but also leads to the increase in the binding strength between metal nanocrystals and carbon support [22]. The fitting spectrum of Co 2p for CoS/CoO@NGNs in Fig. 3b comprises two prominent peaks located at 781.1 and 797.1 eV, corresponding to the 2p_3/2_ and 2p_1/2_, respectively [35]. Two satellite peaks of Co are located at 786.5 and 803.2 eV [45]. In comparison with CoS@NGNs, the Co 2P spectrum of CoS/CoO@NGNs exhibits a positive shift in binding energy, suggesting the electronic coupling and charge transfer between CoS and CoO, in accordance with the DFT prediction. In the S 2p spectrum displayed in Fig. 3c, the peaks at 163.2 and 161.9 eV can be ascribed to S 2p1/2 and S 2p3/2, respectively [46]. The deconvoluted peak at 164.0 eV represents the typical metal–sulfur bond [47], while the subpeak at 162.8 eV originates from S^2−^ [46, 48]. The two peaks at higher binding energy positions correspond to oxidized sulfur [49], which results from partly oxidized sulfur species or oxygen-containing sulfate groups on the surface of the catalyst [50]. It should be mentioned that S atoms may be doped into the NGNs during the hydrothermal sulfurization treatment, which can also impose a positive effect on the ORR/OER activity [51, 52]. The O 1 s XPS spectrum of CoS/CoO@NGNs can be fitted into three subpeaks (Fig. 3d). The O1 peak at 529.5 eV is assigned to the lattice O arising from Co–O bond. The existence of lattice O confirms that CoS is partially reconstructed to CoO during the postannealing process. The O2 peak at 531.6 eV corresponds to surface-absorbed oxygen species (O_2_^2−^/O^−^) and/or hydroxyl groups (−OH) [53]. The O3 peak at 532.2 eV is usually attributed to the oxygen-containing groups (e.g., C–O and COOH) on the surface of the catalyst [53, 54]. Only O3 peak is observed for CoS@NGNs, indicating that O element in CoS@NGNs mainly originates from adsorbed oxygen-containing groups or oxidized S [55].

### ORR and OER Properties

**Fig. 4:**
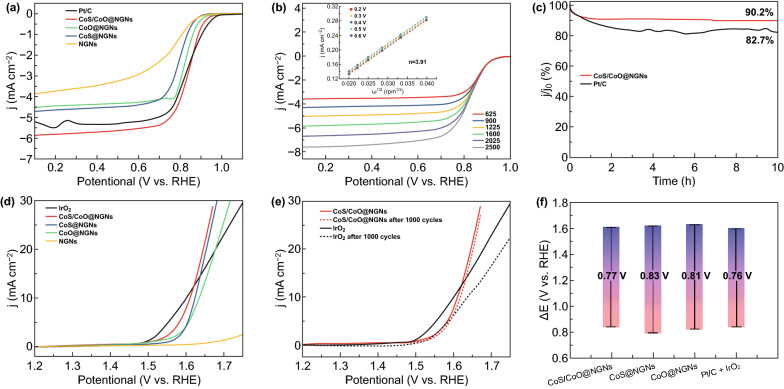
**a** ORR LSV curves of NGNs, CoO@NGNs, CoS@NGNs, CoS/CoO@NGNs and Pt/C catalysts in O_2_-saturated 0.1 M KOH. **b** ORR LSV curves at different rotating speeds and corresponding K–L plots (inset) of CoS/CoO@NGNs. **c** Chronoamperometric responses of CoS/CoO@NGNs and Pt/C catalysts. **d** OER LSV curves of different catalysts in N_2_-saturated 0.1 M KOH. **e** OER LSV curves before and after 1000 CV cycles for CoS/CoO@NGNs and IrO_2_ catalysts. **f** Potential difference between the ORR *E*_1/2_ and OER *E*_j=10_ of different catalysts

**Fig. 5:**
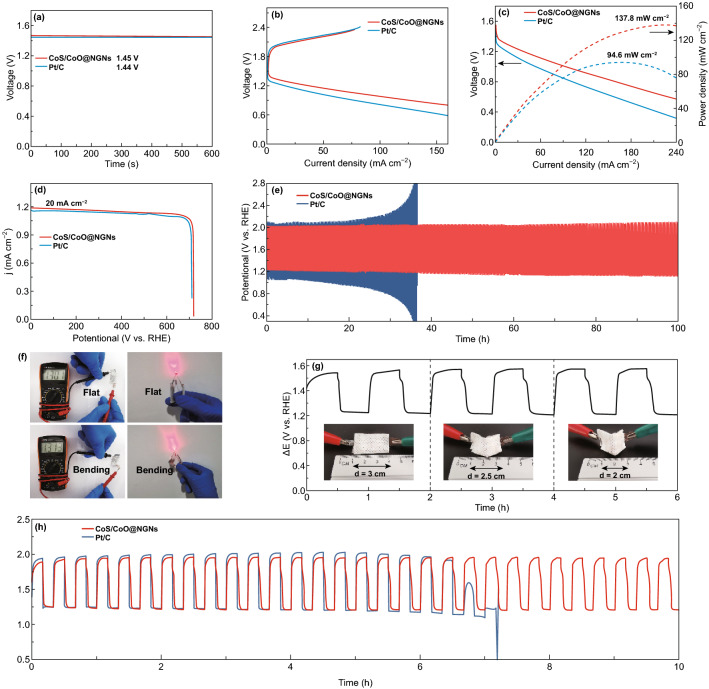
**a** Open-circuit plots of assembled aqueous ZABs. **b** Discharge and charge polarization curves of aqueous ZABs. **c** Corresponding power density plots of aqueous ZABs. **d** Specific capacities of aqueous ZABs at 20 mA cm^−2^. **e** Long-term discharge–charge cycling performances at 10 mA cm^−2^. **f** Photograph of the open-current voltage for the single flexible quasi-solid-state ZAB, and a red LED powered by two batteries connected in series under flat and bending conditions. **g** Discharge–charge cycling curve of the flexible quasi-solid-state ZAB with CoS/CoO@NGNs air electrode under different bending conditions. **h** Discharge–charge cycling curves of flexible quasi-solid-state ZABs at 1 mA cm^−2^

The ORR and OER electrocatalytic properties of the synthesized catalysts were evaluated by a rotating ring disk electrode (RRDE) in 0.1 M KOH. Cyclic voltammetry (CV) was first conducted to investigate the ORR activity of the as-prepared samples. As shown in Fig. S11, the CV curves recorded in N_2_-saturated electrolyte show no cathodic peak. In contrast, a well-defined cathodic peak at 0.82 V (vs. RHE) is observed for CoS/CoO@NGNs in O_2_-saturated 0.1 M KOH, indicating its good capability to catalyze ORR. The linear sweep voltammetry (LSV) polarization curves at a rotation speed of 1600 rpm are shown in Fig. 4a. Remarkably, CoS/CoO@NGNs displays an outstanding ORR activity with a half-wave potential (*E*_1/2_) of 0.84 V (vs. RHE), which is higher than that of CoS@NGNs (*E*_1/2_ = 0.79 V) and CoO@NGNs (*E*_1/2_ = 0.82 V), and equal to that of commercial 20 wt % Pt/C catalyst (*E*_1/2_ = 0.84 V). Moreover, CoO/CoS@NGNs presents the largest reduction current density in the diffusion-controlled region, which means its enhanced mass- and electron-transfer capability resulting from the interfacial structure [10]. The excellent ORR activity of CoS/CoO@NGNs is also manifested in its smaller Tafel slope of 69 mV dec^−1^ (Fig. S12) compared to NGNs (110 mV dec^−1^), CoS@NGNs (73 mV dec^−1^), CoO@NGNs (76 mV dec^−1^) and even Pt/C (75 mV dec^−1^). The catalytic selectivity for ORR was calculated from corresponding disk and ring currents. As shown in Fig. S13a, large disk current densities and much smaller ring current densities are observed for CoS/CoO@NGNs. The calculated results in Fig. S13b show that the HO_2_^–^ yield of CoS/CoO@NGNs is below 5% in the potential range of 0.2–0.8 V. The average electron-transfer number (*n*) is around 3.94, which is equal to that of benchmark Pt/C catalyst. This means a high selectivity of CoS/CoO@NGNs for the four-electron ORR process. The ORR kinetics was further investigated according to the Koutecky–Levich (K–L) equation using LSV curves at different rotation speeds (Figs. 5b and S14). The parallel fitting lines demonstrate the first-order reaction kinetics of CoS/CoO@NGNs regarding the oxygen concentration in the electrolyte (inset of Fig. 4b) [56, 57]. The further calculation reveals that the average transfer number of CoS/CoO@NGNs is about 3.91, in good agreement with the value obtained from the RRDE test. This value is also superior to that of CoS@NGNs (3.52) and CoO@NGNs (3.83) and approaches to that of Pt/C catalyst (3.94). These test results clearly demonstrate that the construction of CoS/CoO interfacial structure can effectively boost the ORR electrocatalytic activity. To evaluate the durability of CoS/CoO@NGNs for ORR, chronoamperometry tests were conducted at 0.4 V (vs. RHE) in O_2_-saturated 0.1 M KOH (Fig. 4c). Remarkably, the current density of CoS/CoO@NGNs remains at 90.2% after 10 h. By contrast, the commercial 20 wt % Pt/C catalyst suffers from a more significant loss of 17.3% in the current density. This comparison illustrates the better durability of CoS/CoO@NGNs than commercial 20 wt% Pt/C catalyst during the ORR process.

The OER performances of the as-prepared catalysts and commercial IrO_2_ were investigated in N_2_-saturated 0.1 M KOH. Figure 4d shows that CoS/CoO@NGNs achieve a current density of 10 mA cm^−2^ at a potential of 1.61 V (*E*_j=10_), whereas CoS@NGNs and CoO@NGNs require the potential of 1.62 and 1.63 V to reach 10 mA cm^−2^, respectively. Notably, the *E*_j=10_ value of CoS/CoO@NGNs is close to that of IrO_2_ (1.60 V). Attributed to its enhanced mass- and electron-transfer capability, the current density of CoS/CoO@NGNs outperforms that of IrO_2_ at higher applied potentials, further demonstrating its outstanding capability to catalyze OER. The OER Tafel plots (Fig. S15) derived from LSV curves reveal that the slope value of CoS/CoO@NGNs (65 mV dec^−1^) is smaller than noble-metal IrO_2_ catalyst (81 mV dec^−1^), demonstrating its faster reaction kinetics. Electrochemical impedance spectroscopy (EIS) tests were conducted to investigate electrochemical impedances of synthesized catalysts for OER. As shown in EIS Nyquist plots (Fig. S16), the smallest semicircle is observed for CoS/CoO@NGNs in the Faradaic reaction domain, indicating its faster electron transfer than that of CoS@NGNs and CoO@NGNs [58]. The reduced electron-transfer impedance of CoS/CoO@NGNs is consistent with the DFT stimulation and confirms that the construction of CoS/CoO interface can improve the intrinsic conductivity. To demonstrate the superiority of the interface, we also physically mixed CoS@NGNs and CoO@NGNs with the mass ratio of 1:1 and evaluated the corresponding ORR and OER catalytic performances. As depicted in Fig. S17, the catalytic activity of the mixed catalyst is much inferior to CoS/CoO@NGNs, further suggesting the importance of interfaces in enhancing catalytic activity for both ORR and OER.

The durability for OER was assessed by the long-term CV test. As presented in Fig. 4e, CoS/CoO@NGNs catalyst shows a slight degradation with the *E*_j=10_ shifting to 1.62 V after 1000 cycles. In comparison, IrO_2_ goes through significant performance fading (*E*_j=10_ = 1.64 V after 1000 cycles). These results demonstrate the better OER catalytic stability of CoS/CoO@NGNs. The impressive electrocatalytic durability of CoS/CoO@NGNs can be ascribed to the strong interaction between CoS/CoO nanocrystals and NGNs, which inhibits the aggregation and migration of active species during oxygen electrocatalysis [33]. The electrochemical double-layer capacitance (*C*_dl_) analysis was performed to evaluate the electrochemical surface area (ECSA) of synthesized catalysts (Fig. S18). The CoS/CoO@NGNs catalyst delivers a higher *C*_dl_ value of 21.6 mF cm^−2^ than that of CoS@NGNs (16.8 mF cm^−2^) and CoO@NGNs (17.9 mF cm^−2^), suggesting that more active sites are formed through constructing heterogeneous CoS/CoO nanocrystals, in line with the better ORR and OER performances of the CoS/CoO@NGNs catalyst. The potential difference between *E*_j=10_ for OER and *E*_1/2_ for ORR (Δ*E *= *E*_j=10_ − *E*_1/2_) is calculated to assess the bifunctional activity of synthesized samples. The result is illustrated in Fig. 4f. The CoS/CoO@NGNs present a small Δ*E* value of 0.77 V, which is close to that of commercial Pt/C and IrO_2_ (0.76 V), demonstrating its outstanding bifunctional activity for both ORR and OER. The Δ*E* value of CoS/CoO@NGNs in this work is also comparable to other advanced bifunctional ORR/OER electrocatalysts reported recently, as seen in Table S1.

### Rechargeable Zn–Air Battery Performances

To evaluate the practical application of CoS/CoO@NGNs, the aqueous ZABs were first assembled by using CoS/CoO@NGNs as the cathode. The Pt/C + IrO_2_ mixture catalyst with the mass ratio of 1:1 was also tested for comparison. All the battery tests were conducted in ambient air. As shown in Fig. 5a, the open-circuit voltage of ZAB with CoS/CoO@NGNs cathode (1.45 V) is similar to that of noble-metal-based battery (1.44 V). The polarization curves in Fig. 5b reveal a smaller charge–discharge voltage gap of CoS/CoO@NGNs air electrode than that of the Pt/C + IrO_2_ air electrode at the same current density, suggesting a better ORR/OER activity of CoS/CoO@NGNs air electrode. The corresponding peak power density of CoS/CoO@NGNs is 137.8 mW cm^−2^ (Fig. 5c), surpassing that of Pt/C + IrO_2_ (94.6 mW cm^−2^). Furthermore, the CoS/CoO@NGNs air electrode displays a higher voltage platform than that of Pt/C + IrO_2_ air electrode at a discharge current of 20 mA cm^−2^. High specific capacity of 723.9 mAh g^−1^ can be achieved (Fig. 5d), corresponding to a gravimetric energy density of 832.5 Wh kg^−1^. These parameters are superior to the commercial noble-metal battery (specific capacity = 711.1 mAh g^−1^, energy density = 800.5 Wh kg^−1^), further demonstrating its better ORR activity. To evaluate the cycling stability, the battery was cycled at 10 mA cm^−2^. As shown in Fig. 5e, the initial discharge and charge voltages of CoS/CoO@NGNs-based battery are 1.23 and 2.01 V, respectively, contributing to an outstanding round-trip efficiency of 61.2% (Fig. 5e). For comparison, the noble-metal Pt/C + IrO_2_-based battery displays an initial discharge voltage of 1.18 V and charge voltage of 2.07 V with a low round-trip efficiency of 57.0%. The as-assembled Zn-air battery with the CoS/CoO@NGNs composite exhibits comparable peak power density and cycling efficiency to the catalysts in the references (Table S2). After continuous cycling for 100 h, the gentle voltage change is observed for the CoS/CoO@NGNs air electrode. In comparison, the voltage gap of the Pt/C + IrO_2_-based battery increases gradually only after 20 h. This demonstrates the better cycling durability of CoS/CoO@NGNs. Due to the lack of strong metal–support interaction, the dissolution or aggregation of the noble-metal nanoparticles may occur during the electrocatalytic reaction, thus leading to severe performance degradation [59].

After the long-term discharge–charge cycling test, the crystal structure and morphology of CoS/CoO@NGNs were further examined. The XRD pattern in Fig. S19 shows that the characteristic diffraction peaks of CoS and CoO are still preserved and no additional impurity peak is observed compared with pristine CoS/CoO@NGNs. The TEM image in Fig. S20a reveals that the spatial dispersion of CoS/CoO nanocrystals on the surface of NGNs is well maintained, further verifying the strong coupling between metal species and NGNs substrate, consistent with DFT simulations. Moreover, as depicted in the HRTEM image (Fig. S20b), the interface and lattice disorders still exist in CoS/CoO@NGNs after long-term discharge–charge cycling. These results further reveal the excellent structural integrity and catalytic durability of CoS/CoO@NGNs as the air electrode in rechargeable ZABs.

The flexible quasi-solid-state ZABs were further assembled with CoS/CoO@NGNs air electrode and alkaline polyvinyl alcohol (PVA) gel electrolyte. As illustrated in Fig. 5f, under both flat and bending states, the single quasi-solid-state battery can deliver a stable open-circuit potential of 1.3 V in ambient air, and two batteries connected in series can easily power a red light-emitting diode (LED, 2.2 V), suggesting its excellent mechanical flexibility. The discharge and charge polarization curves of the flexible quasi-solid-state ZABs are presented in Fig. S21. The battery with CoS/CoO@NGNs still demonstrates a narrower voltage gap and higher power density (39.3 mW cm^−2^) than these of battery with Pt/C + IrO_2_ (36.2 mW cm^−2^). The mechanical flexibility of the battery is further demonstrated by the uninterrupted discharge–charge cycling curve under different bending conditions (Fig. 5g). Meanwhile, during a 10-h test at 1 mA cm^−2^, the flexible quasi-solid-state ZAB with CoS/CoO@NGNs air electrode displays fewer fluctuations in the discharge and charge voltage than the Pt/C + IrO_2_ air electrode (Fig. 5h), indicating the excellent bifunctional ORR/OER activity of CoS/CoO@NGNs even in a gel electrolyte. These results demonstrate the great potential of CoS/CoO@NGNs for applications in wearable and flexible electronic devices.

## Conclusions

In summary, a nanocomposite material with well-defined interfacial structures of heterogeneous CoS/CoO nanocrystals supported on N-doped graphene was fabricated through rational interfacial regulation. DFT calculations demonstrated that the boundary interface between CoS and CoO could induce a charge transfer effect, leading to modulated electronic structure and enhanced electronic conductivity. Meanwhile, the strong interaction between CoS/CoO and N-doped graphene can further accelerate the electron transfer and guarantee the excellent stability of the catalyst. As a noble-metal free bifunctional electrocatalyst, the synthesized CoS/CoO@NGNs catalyst presents enhanced electrocatalytic activity and outstanding stability toward both ORR and OER. Moreover, CoS/CoO@NGNs cathode exhibits excellent rechargeability in real battery tests. This work provides a facile and efficient strategy for the rational design of high-performance bifunctional electrocatalysts for rechargeable ZABs via interface engineering.

## Electronic supplementary material

Below is the link to the electronic supplementary material.

Supplementary material 1 (DOC 14172 kb)
