# Correction to: Interface Engineering of CoS/CoO@N‑Doped Graphene Nanocomposite for High‑Performance Rechargeable Zn–Air Batteries

**DOI:** 10.1007/s40820-021-00608-4

**Published:** 2021-03-23

**Authors:** Yuhui Tian, Li Xu, Meng Li, Ding Yuan, Xianhu Liu, Junchao Qian, Yuhai Dou, Jingxia Qiu, Shanqing Zhang

**Affiliations:** 1 Institute for Energy Research, School of Chemistry and Chemical Engineering, Key Laboratory of Zhenjiang, Jiangsu University, Zhenjiang, 212013 People’s Republic of China; 2 Centre for Clean Environment and Energy, School of Environment and Science, Gold Coast Campus, Griffith University, Gold Coast, QLD 4222 Australia; 3 Key Laboratory of Materials Processing and Mold (Zhengzhou University), Ministry of Education, Zhengzhou, People’s Republic of China; 4 Jiangsu Key Laboratory for Environment Functional Materials, Suzhou University of Science and Technology, Suzhou, 215009 People’s Republic of China

## Correction to: Nano-Micro Lett. (2021) 13:3 https://doi.org/10.1007/s40820-020-00526-x

In the original publication, the label text “Pt/C” in Fig. 5 should be “Pt/C + IrO_2_”. In Fig. 5d, the X-axis label “Potential (V vs. RHE)” should be replaced with “Specific capacity (mAh g^−1^)”. In Fig. 5e, the Y-axis label “Potential (V vs. RHE)” should be replaced with “Voltage (V)”. In Fig. 5g, the X-axis label “Time (h)” should be replaced with “Cycle number (n)”. The Y-axis label “Δ*E* (V vs. RHE)” should be replaced with “Voltage (V)”. The number “1.4” and “1.6” should be replaced with 1.6 and 2.0, respectively. The corresponding data analysis and conclusions in the manuscript are not affected and thus not to be changed. The correct Fig. 5 is provided in this correction.

**Fig. 5:**
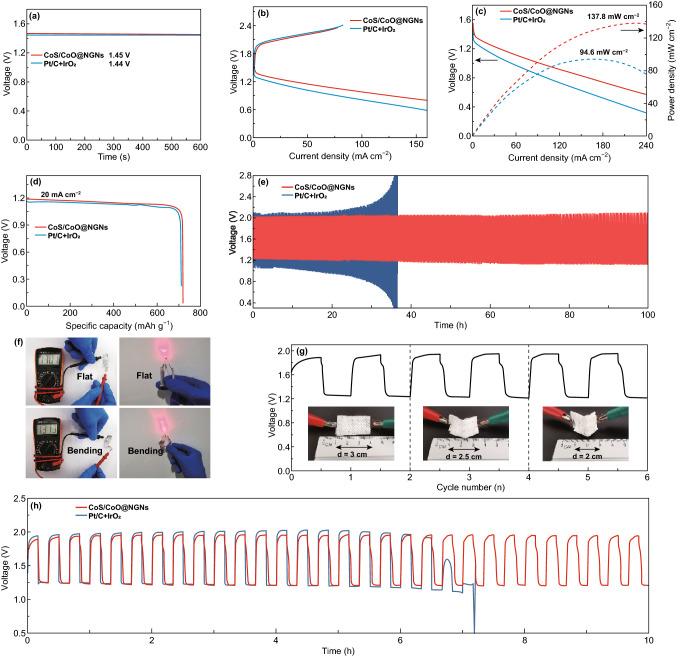
Open circuit plots of assembled aqueous ZABs. **b** Discharge and charge polarization curves of aqueous ZABs. **c** The corresponding power density plots of aqueous ZABs. **d** Specific capacities of aqueous ZABs at 20 mA cm^−2^. **e** Long-term discharge–charge cycling performances at 10 mA cm^−2^. **f** Photograph of the open-current voltage for the single flexible quasi-solid-state ZAB, and a red LED powered by two batteries connected in series under flat and bending conditions. **g** The discharge–charge cycling curve of the flexible quasi-solid-state ZAB with CoS/CoO@NGNs air electrode under different bending conditions. **h** Discharge–charge cycling curves of flexible quasi-solid-state ZABs at 1 mA cm^−2^

