# Supplementary material for: Interface Engineering of CoS/CoO@N-Doped Graphene Nanocomposite for High-Performance Rechargeable Zn–Air Batteries
Source: Nanomicro Lett. 2020 Oct 27;13:3. doi: 10.1007/s40820-020-00526-x (PMC7988027; doi:10.1007/s40820-020-00526-x)
Supplement: Supplementary file 1 — Supplementary material 1 (DOC 14172 kb) [file 40820_2020_526_MOESM1_ESM.doc]

**Supporting Information**

**Interface Engineering of CoS/CoO@N-Doped Graphene Nanocomposite for High-Performance Rechargeable Zn–Air Batteries**

Yuhui Tian^1,2^, Li Xu^1,2^*, Meng Li^2^, Ding Yuan^1,2^, Xianhu Liu^3^, Junchao Qian^4^, Yuhai Dou^2^, Jingxia Qiu^1^, Shanqing Zhang^1,2^*

^1^ Institute for Energy Research, School of Chemistry and Chemical Engineering, Key Laboratory of Zhenjiang, Jiangsu University, Zhenjiang 212013, P. R. China

^2^ Centre for Clean Environment and Energy, School of Environment and Science, Gold Coast Campus, Griffith University, QLD 4222, Australia

^3^ Key Laboratory of Materials Processing and Mold (Zhengzhou University), Ministry of Education, Zhengzhou, P.R. China,

^4^ Jiangsu Key Laboratory for Environment Functional Materials, Suzhou University of Science and Technology, Suzhou 215009, P. R. China

*Corresponding authors.

E-mail addresses:

[xulichem@ujs.edu.cn](mailto:xulichem@ujs.edu.cn) (L. Xu)

[s.zhang@griffith.edu.au](mailto:s.zhang@griffith.edu.au) (S.Q. Zhang)

**Experimental Section**

**Chemicals**

All the chemical reagents were used without further treatment. Cobalt (Ⅱ) nitrate hexahydrate (Co(NO_3_)_2_·6H_2_O), hexamethylenetetramine, thioacetamide, ethylene glycol, potassium hydroxide, and ethanol (99.7%) were purchased from Sinopharm Chemical Reagent Co., Ltd. Graphene oxide (GO) powder was obtained from Nanjing XFNANO Materials Tech Co., Ltd. Nafion solution (10 wt%) was purchased from Sigma-Aldrich. Commercial Pt/C (20 wt%) catalyst was obtained from Alfa Aesar Chemicals Co., Ltd.

**Synthesis of the Co(OH)_2_ nanosheets**

In the typical synthesis, Co(NO_3_)_2_·6H_2_O (1 mmol) and hexamethylenetetramine (2 mmol) were dissolved in 25 mL of deionized water and ethylene glycol (with a volume ratio of 1:1.5). After stirring for 10 min, the above solution was transferred into a 25 mL Teﬂon-lined stainless-steel autoclave and heated at 120 °C for 6 h. After cooling down to room temperature, the product was obtained by rinsing several times with deionized water and ethanol and dried in a vacuum oven at 60 °C overnight.

**Synthesis of the N-doped graphene nanosheets**

The GO (100 mg) powder was placed into a tube furnace with a ramping rate of 10 °C min^−1^ under argon flow. When the temperature reached 800 °C, the argon flow was replaced by ammonia flow for 1 h. The sample was then cooled to room temperature under the argon atmosphere. The resulting sample was denoted as NGNs.

**Physical Characterization**

Transmission electron microscopy (TEM, JEOL JEM-2010F) and field-emission scanning electron microscopy (SEM, JEOL JSM-7800F) were utilized to evaluate the morphology of the prepared catalysts. The crystal structures were characterized by X-ray diffraction (Shimadzu, XRD-6100) using high-intensity Cu Kα radiation source (λ=1.54 Å) and operating at a voltage of 40 kV and current of 30 mA. X-ray photoelectron spectroscopy (XPS) analysis was performed using a Thermo ESCALB 250XI X-ray photoelectron spectrometer. Specific surface areas and pore size distributions were obtained from nitrogen sorption isotherms at 77 K (Micromeritics Instrument Corporation, USA) by using Brunauer−Emmett−Teller and Barrett−Joyner−Halenda (BJH) methods, respectively.

**Fig. S1** The top view of **a** N-doped graphene layer, and **b** N-doped graphene supported CoS. The brown, silver, blue, and red balls represent C, N, O, and Co atoms, respectively.

**Fig. S2** The top view of **a** N-doped graphene layer, and **b** N-doped graphene supported CoO. The brown, silver, blue, and red balls represent C, N, O, and Co atoms, respectively.

**Fig. S3** Contour plots of differential charge density of **a** CoS-NG, and **b** CoO-NG model. The yellow and cyan regions represent the charge accumulation and charge depletion, respectively. The isosurface level was set to be 0.015 eÅ^–1^.

**Fig. S4 a** XRD pattern, and **b** SEM image of Co(OH)_2_ nanosheets.

**Fig. S5 a** XRD pattern, and **b** SEM image of NGNs.

**Fig. S6 a** XRD pattern, and **b** SEM image of CoS@NGNs.

**Fig. S7 a** XRD pattern, and **b** SEM image of CoO@NGNs.

**Fig. S8** The electron energy loss spectroscopy (EELS) line-scan profile of the corresponding CoS/CoO nanocrystal (inset: high-angle annular dark-field scanning transmission electron microscope (HAADF-STEM) image of isolated CoS/CoO nanocrystal).

**Fig. S9** N_2_ adsorption-desorption isotherms of **a** NGNs, and **c** CoS/CoO@NGNs. Corresponding pore size distributions of **b** NGNs, and **d** CoS/CoO@NGNs.

**Fig. S10** XPS full-range spectrum of CoS/CoO@NGNs.

**Fig. S11** CV curves of CoS/CoO@NGNs, CoO@NGNs, CoS@NGNs, and NGNs in N_2_ (dotted line) or O_2_ (solid line) saturated 0.1 M KOH electrolyte.

**Fig. S12** ORR Tafel plots of NGNs, CoO@NGNs, CoS@NGNs, CoS/CoO@NGNs, and Pt/C catalysts recorded at 1600 rpm.

**Fig. S13** **a** RRDE disk and ring current, **b** corresponding HO_2_^–^ yield, and electron transfer number per O_2_ during the ORR process for CoS/CoO@NGNs and Pt/C catalysts.

**Fig. S14** ORR LSV curves at different rotating speeds and corresponding K–L plots (inset) of **a-b** commercial Pt/C, **c-d** CoO@NGNs and **e-f** CoS@NGNs catalysts.

**Fig. S15** OER Tafel plots of NGNs, CoO@NGNs, CoS@NGNs, CoS/CoO@NGNs, and IrO_2_ catalysts recorded at 1600 rpm.

**Fig. S16** Nyquist plots of CoS/CoO@NGNs, CoS@NGNs, and CoO@NGNs catalysts in N_2_-saturated 0.1 M KOH at 1.6 V (vs. RHE).

**Fig. S17** **a** ORR and **b** OER LSV curves of CoS/CoO@NGNs and physically mixed CoS@NGNs + CoO@NGNs catalysts.

**Fig. S18 a**, **c**, and **e** CV curves with different scan rates (2, 5, 10, 15, 20, 25, and 50 mV s^–1^) of CoS/CoO@NGNs, CoO@NGNs, CoS@NGNs, and NGNs in 0.1 M KOH. **b**, **d**, and **f** The corresponding difference of current density at 1.01 V (vs. RHE).

**Fig. S19** XRD pattern of CoS/CoO@NGNs air electrode after the cycling test.

**Fig. S20** **a** TEM and **b** HRTEM images of CoS/CoO@NGNs after the cycling test.

**Fig. S21** Discharge and charge polarization curves of CoS/CoO@NGNs and Pt/C+IrO_2_-based flexible quasi-solid-state ZABs.

**Table S1.** Comparison of ORR and OER activity parameters for cobalt-based bifunctional catalysts reported in the literature.

| **Catalysts** | ***E*_1/2_ (V)** | ***E*_j=10_ (V)** | **Δ*E* (V)** | **Ref** |
| --- | --- | --- | --- | --- |
| CoS/CoO@NSNGs | 0.84 | 1.61 | 0.77 | This work |
| CoS@NSNGs | 0.79 | 1.62 | 0.83 | This work |
| CoO@NSNGs | 0.82 | 1.63 | 0.81 | This work |
| Pt/C | 0.84 | - |  | This work |
| IrO_2_ | - | 1.60 |  | This work |
| Co@Co_3_O_4_/NC | 0.80 | 1.68 | 0.84 | [1] |
| Co_9_S_8_/NSC-900 | 0.88 | 1.64 | 0.76 | [2] |
| Co_9_S_8_/NSPG-900 | 0.800 | 1.573 | 0.773 | [3] |
| CoO_0.87_S_0.13_/GN | 0.83 | 1.59 | 0.76 | [4] |
| Co_9_S_8_/CNT | 0.82 | 1.599 | 0.779 | [5] |
| CoS_x_@PSN/rGO | 0.78 | 1.57 | 0.79 | [6] |
| Ni-MnO/rGO | 0.78 | 1.60 | 0.82 | [7] |
| MnO/Co/PGC | 0.78 | 1.60 | 0.82 | [8] |
| N-Co_3_O_4_@NC-2 | 0.77 | 1.55 | 0.78 | [9] |
| In-CoO/CoP FNS | 0.81 | 1.597 | 0.787 | [10] |

**Table S2.** The comparison between the battery performances of this works and the works in the literature.

| **Catalysts** | **Peak power density**  **(mW cm**^−^**^2^)** | **Discharge/**  **Charge current density**  **(mA cm**^−^**^2^)** | **Discharge/charge potential gap (V)** | **Corresponding energy efficiency (%)** | **Ref** |
| --- | --- | --- | --- | --- | --- |
| CoS/CoO@NGNs | 137.8 | 10 | 0.78 | 61.2 | This work |
| FeP/Fe_2_O_3_@NPCA | 130 | 10 | - | 52.17 | [11] |
| PB@Met-700 | 148 | 10 | - | ≈57.57 | [12] |
| CoS_x_/Co-NC-800 | 103 | 2 | 0.73 | 62.9 | [13] |
| NiCo_2_S_4_@g-C_3_N_4_-CNT | 163 | 10 | 0.84 | 60.6 | [14] |
| IOSHs-NSC-Co_9_S_8_ | 133 | 10 | 0.872 | 57.5 | [15] |
| N-Co_3_O_4_@NC-2 | 174.1 | 5 | 0.80 | 58.6 | [16] |
| MnO/Co/PGC | 172 | 10 | - | 59 | [17] |
| Co_9_S_8_/NSG‑700 | 72.14 | 10 | 0.86 | - | [18] |
| BCZ2 | - | 5 | 0.83 | - | [19] |

**References**

1. A. Aijaz, J. Masa, C. Rösler, W. Xia, P. Weide, A.J. Botz, R.A. Fischer, W. Schuhmann, M. Muhler, Co@Co_3_O_4_ encapsulated in carbon nanotube-grafted nitrogen-doped carbon polyhedra as an advanced bifunctional oxygen electrode. Angew. Chem. Int. Ed. **55**(12), 4087-4091 (2016). https://doi.org/10.1002/anie.201509382

2. Z.Q. Cao, M.Z. Wu, H.B. Hu, G.J. Liang, C.Y. Zhi, Monodisperse Co_9_S_8_ nanoparticles in situ embedded within N, S-codoped honeycomb-structured porous carbon for bifunctional oxygen electrocatalyst in a rechargeable Zn–air battery, NPG Asia Mater. **10**(7), 670-684 (2018). https://doi.org/10.1038/s41427-018-0063-0

3. X.X. Ma, X.H. Dai, X.Q. He, Co_9_S_8_-modified N, S, and P ternary-doped 3D graphene aerogels as a high-performance electrocatalyst for both the oxygen reduction reaction and oxygen evolution reaction. ACS Sustainable Chem. Eng. **5**(11), 9848-9857 (2017). https://doi.org/10.1021/acssuschemeng.7b01820

4. J. Fu, F.M. Hassan, C. Zhong, J. Lu, H. Liu, A.P. Yu, Z.W. Chen, Defect engineering of chalcogen-tailored oxygen electrocatalysts for rechargeable quasi-solid-state zinc-air batteries, Adv. Mater. **29**(35), 1702526 (2017). https://doi.org/10.1002/adma.201702526

5. H. Li, Z. Guo, X.W. Wang, Atomic-layer-deposited ultrathin Co_9_S_8_ on carbon nanotubes: An efficient bifunctional electrocatalyst for oxygen evolution/reduction reactions and rechargeable Zn–air batteries. J. Mater. Chem. A. **5**(40), 21353-21361 (2017). https://doi.org/10.1039/c7ta06243e

6. W.H. Niu, Z. Li, K. Marcus, L. Zhou, Y.L. Li, R.Q. Ye, K. Liang, Y. Yang, Surface-modified porous carbon nitride composites as highly efficient electrocatalyst for Zn-air batteries. Adv. Energy Mater. **8**(1), 1701642 (2017). https://doi.org/10.1002/aenm.201701642

7. G.T. Fu, X.X. Yan, Y.F. Chen, L. Xu, D.M. Sun, J.M. Lee, Y.W. Tang, Boosting bifunctional oxygen electrocatalysis with 3d graphene aerogel-supported Ni/MnO particles. Adv. Mater. **30**(5), 1704609 (2017). https://doi.org/10.1002/adma.201704609

8. X.F. Lu, Y. Chen, S. Wang, S.Y. Gao, X.W. Lou, Interfacing manganese oxide and cobalt in porous graphitic carbon polyhedrons boosts oxygen electrocatalysis for Zn-air batteries. Adv. Mater. **31**(39), 1902339 (2019). https://doi.org/10.1002/adma.201902339

9. Z.C. Wang, W.J. Xu, X.K. Chen, Y.H. Peng, Y.Y. Song, C.X. Lv, H.L. Liu, J.W. Sun, D. Yuan, X.Y. Li, X.X. Guo, D.J. Yang, L.X. Zhang, Defect-rich nitrogen doped Co_3_O_4_/C porous nanocubes enable high-efficiency bifunctional oxygen electrocatalysis. Adv. Funct. Mater. **29**(33), 1902875 (2019). https://doi.org/10.1002/adfm.201902875

10. W. Jin, J.P. Chen, B. Liu, J.G. Hu, Z.X. Wu, W.Q. Cai, G.T. Fu, Oxygen vacancy-rich in-doped CoO/CoP heterostructure as an effective air cathode for rechargeable Zn-air batteries. Small **15**(46), 1904210 (2019). <https://doi.org/10.1002/smll.201904210>

11. K. Wu, L. Zhang, Y. Yuan, L. Zhong, Z. Chen, X. Chi, H. Lu, Z. Chen, R. Zou, T. Li, C. Jiang, Y. Chen, X. Peng, J. Lu, An iron-decorated carbon aerogel for rechargeable flow and flexible Zn-air batteries. Adv. Mater. 2002292 (2020). <https://doi.org/10.1002/adma.202002292>

12. Y. Lian, K. Shi, H. Yang, H. Sun, P. Qi, J. Ye, W. Wu, Z. Deng, Y. Peng, Elucidation of active sites on s, n codoped carbon cubes embedding co-fe carbides toward reversible oxygen conversion in high-performance zinc-air batteries. Small **16**(23), 1907368 (2020). https://doi.org/10.1002/smll.201907368

13. Q. Lu, J. Yu, X. Zou, K. Liao, P. Tan, W. Zhou, M. Ni, Z. Shao, Self-catalyzed growth of Co, N-codoped CNTs on carbon-encased CoS*_x_* surface: A noble-metal-free bifunctional oxygen electrocatalyst for flexible solid Zn–air batteries. Adv. Funct. Mater. **29**(38), 1904481 (2019). https://doi.org/10.1002/adfm.201904481

14. X. Han, W. Zhang, X. Ma, C. Zhong, N. Zhao, W. Hu, Y. Deng, Identifying the activation of bimetallic sites in NiCo_2_S_4_@g-C_3_N_4_-CNT hybrid electrocatalysts for synergistic oxygen reduction and evolution. Adv. Mater. **31**(18) 1808281 (2019). https://doi.org/10.1002/adma.201808281

15. K. Tang, C. Yuan, Y. Xiong, H. Hu, M. Wu, Inverse-opal-structured hybrids of N, S-codoped-carbon-confined Co_9_S_8_ nanoparticles as bifunctional oxygen electrocatalyst for on-chip all-solid-state rechargeable Zn-air batteries. Appl. Catal. B **260** 118209 (2020). https://doi.org/10.1016/j.apcatb.2019.118209

16. Z. Wang, W. Xu, X. Chen, Y. Peng, Y. Song, C. Lv, H. Liu, J. Sun, D. Yuan, X. Li, X. Guo, D. Yang, L. Zhang, Defect‐rich nitrogen doped Co_3_O_4_/C porous nanocubes enable high‐efficiency bifunctional oxygen electrocatalysis. Adv. Funct. Mater. **29**(33), 1902875 (2019). <https://doi.org/10.1002/adfm.201902875>

17. X.F. Lu, Y. Chen, S. Wang, S. Gao, X.W. Lou, Interfacing manganese oxide and cobalt in porous graphitic carbon polyhedrons boosts oxygen electrocatalysis for Zn-air batteries. Adv. Mater. **31**(39), 1902339 (2019). <https://doi.org/10.1002/adma.201902339>

18. Q. Shao, J. Liu, Q. Wu, Q. Li, H.-g. Wang, Y. Li, Q. Duan, In situ coupling strategy for anchoring monodisperse Co_9_S_8_ nanoparticles on S and N dual-doped graphene as a bifunctional electrocatalyst for rechargeable Zn–air battery. Nano-Micro Lett. **11**(1), (2019). https://doi.org/10.1007/s40820-018-0231-3

19. Y. Arafat, M.R. Azhar, Y. Zhong, X. Xu, M.O. Tadé, Z. Shao, A porous nano-micro-composite as a high-performance bi-functional air electrode with remarkable stability for rechargeable zinc–air batteries. Nano-Micro Lett. **12**(1), (2020). <https://doi.org/10.1007/s40820-020-00468-4>
